# A Novel Targeted Screening Tool for Hypogammaglobulinemia: Measurement of Serum Immunoglobulin (IgG, IgM, IgA) Levels from Dried Blood Spots (Ig-DBS Assay)

**DOI:** 10.1007/s10875-015-0184-y

**Published:** 2015-08-01

**Authors:** Leman Yel, Christopher J. Rabbat, Charlotte Cunningham-Rundles, Jordan S. Orange, Troy R. Torgerson, James W. Verbsky, Yeong Wang, Maoyong Fu, Terry S. Robins, Marc S. Edwards, Jesper Nymann-Andersen

**Affiliations:** 1 Baxalta US, Inc., Global Clinical Research, One Baxter Way, Westlake Village, CA 91362 USA; 2 University of California, Irvine, CA USA; 3 Baxalta US, Inc., North American Medical Affairs, Bannockburn, IL USA; 4 Mount Sinai School of Medicine, New York, NY USA; 5 Texas Children’s Hospital, Baylor College of Medicine, Houston, TX USA; 6 University of Washington and Seattle Children’s Hospital, Seattle Children’s Research Institute, Seattle, WA USA; 7 Medical College of Wisconsin, Milwaukee, WI USA; 8 Baxalta US, Inc., Global Quality, Westlake Village, CA USA; 9 Quest Diagnostics, Valencia, CA USA

**Keywords:** DBS, dried blood spots, ELISA, Ig-DBS assay, immunoglobulin, nephelometry, primary immunodeficiency, validation

## Abstract

**Purpose:**

To develop an assay to quantify serum immunoglobulin (IgG, IgM, IgA) levels using dried blood spots (DBS) obtained on collection cards to be used as a tool for targeted screening for hypogammaglobulinemia.

**Methods:**

DBS samples, along with simultaneous serum samples, were collected from 107 healthy individuals (11 months to 57 years of age). After eluting proteins from DBS, IgG, IgM, and IgA were quantified by an enzyme-linked immunosorbent assay (ELISA). The Ig-DBS assay was validated through calibration curve performance, intra- and inter-assay precision, accuracy, specificity, selectivity, and linearity. The ELISA measurements were compared with serum Ig levels obtained using a standard nephelometry assay on serum samples collected simultaneously with the DBS samples and the results of the two assays were correlated. The stability of IgG, IgM, and IgA in the DBS was tested at room temperature, 36° to 38 °C, 2 to 8 °C, and −25 to −40 °C, from 4 to 14 days.

**Results:**

The Ig-DBS assay demonstrated precision, accuracy, specificity, selectivity, and linearity. Using the identified correlation coefficients of 0.834 for IgG, 0.789 for IgM, and 0.918 for IgA, the standard nephelometry-based normal reference ranges for all 3 serum Ig isotypes could be used with the Ig-DBS assay in individuals ≥16 years of age. The DBS samples were stable for 14 days at room temperature in a closed polyethylene bag.

**Conclusions:**

The Ig-DBS assay is both sensitive and accurate for quantification of serum immunoglobulins. Samples are sufficiently stable at ambient temperature to allow for convenient shipping and analysis at a centralized laboratory. This assay therefore presents a new option for screening patients ≥16 years of age for hypogammaglobulinemia in any setting.

## Introduction

Primary immunodeficiency diseases (PIDs) are a heterogeneous group of more than 200 disorders that result from a large number of different genetic abnormalities affecting the innate and adaptive immune systems [1]. It has been estimated that there are about 250,000 patients in the United States who have been diagnosed with PID, and many others still to be identified [2]. Worldwide, PIDs are more common than generally thought [3, 4]. Antibody deficiencies account for more than half (50.9 %) of all immunodeficiency disorders [4]. This heterogeneous group of disorders is characterized by impaired antibody production leading to low quantity and/or quality of antibodies [5]. Unfortunately, these disorders are likely to go undiagnosed for years, delaying initiation of appropriate therapy [6].

Many types of PID involving antibody deficiency can be effectively managed by administering immunoglobulin (Ig). In this context, Ig therapy has been shown to significantly decrease the risk of infections and improve quality of life [7–10]. Early diagnosis and treatment lead to better clinical outcomes with decreased risk for morbidity and mortality [6, 11–14].

To improve early diagnosis of PID, several initiatives starting with awareness campaigns have been introduced [15, 16]. Development of newborn screening programs for severe combined immunodeficiency (SCID) changed the paradigm for detecting PID from that of identifying patients after they suffer infectious complications from their disease to that of prospectively screening large populations before the manifestations of their disease become clinically apparent [17–23]. Currently, the US Department of Health and Human Services recommends screening for SCID in all newborns by detecting T cell receptor excision circles (TREC) [24]. The platform for TREC testing employs the dried blood spot obtained from neonates for the purpose of congenital disease testing. This provides the flexibility in obtaining the sample as well as uniform access. The detection of severe B cell disorders, such as X-linked agammaglobulinemia, has also been proposed through this vehicle through detection of the B cell receptor genetic recombination product [25, 26].

While these approaches can and do detect the most severe forms of PID, the vast majority of conditions and patients will not be identified via these modalities.

To raise awareness and increase the index of suspicion for PID in general, various lists of suggestive signs and symptoms have been proposed [2, 27–29]. These include recurrent respiratory tract infections (otitis media, sinusitis, bronchitis, pneumonia), gastrointestinal infections, recurrent/chronic diarrhea, meningitis and/or sepsis, and autoimmunity [2]. Some patients having these manifestations may have hypogammaglobulinemia. Because immunoglobulin levels are abnormal in many PIDs, it is recommended that the initial evaluation of such patients should start with quantitation of serum IgG, IgM, and IgA [2, 6, 30].

Nevertheless, the under-diagnosis and substantial diagnostic delays in identifying antibody deficiencies suggest that there continues to be significant barriers to obtaining this important screening test early in the course of disease. In an attempt to decrease one barrier to screening, we developed a simpler targeted screening tool that can be implemented in primary care or specialty clinics to facilitate the timely diagnosis of hypogammaglobulinemia. To do this, we developed a dried blood spot assay for measurement of serum Ig levels that correlates with results obtained using the standard nephelometry assay, remains stable during transport by regular mail service, and permits the collection of samples in any clinical setting.

## Methods

### Samples

**Fig. 1:**
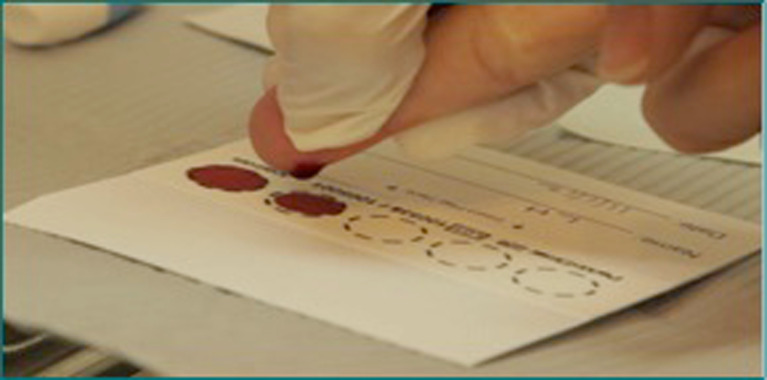
Blood sample collection using a DBS card. Photo, IDBS spot saver card, ID biological systems, PerkinElmer, Greenville, SC, USA

All samples were drawn from 107 healthy individuals (50 males and 57 females) ranging in age from 11 months to 57 years, each of whom or their parents/guardians signed consent forms. Blood samples (both venous blood draws and DBS) were obtained simultaneously at the same visit from healthy individuals. Drops of blood were applied to collection cards (IDBS Spot Saver Card, ID Biological Systems, Greenville, SC, USA) (Fig. 1) after performing finger capillary puncture using a lancet. The circles on the collection card were entirely saturated. The collection cards were allowed to dry on a flat surface for approximately 3 h at room temperature (18 °C to 25 °C), and then were transported to the laboratory inside a polyethylene bag that was contained in a sealed envelope.

### Immunoglobulin Elution from DBS Cards

The DBS specimen was inspected for proper collection and sample integrity. The sample was punched with a Harris Uni-Core puncher and placed into a 1.5 mL low protein binding Eppendorf tube with 200 μL of elution buffer (phosphate-buffered saline [PBS], 0.05 % Tween-20), and incubated at room temperature for 2 h on a shaker at 1000 to 1200 RPM. It was then cooled to 2 °C to 8 °C and shaken for 16 to 18 h at 1000 to 1200 RPM. The eluate was allowed to reach room temperature and then was either used within 4 h or frozen at −20 °C to be used later within 1 month.

### Quantification of IgG, IgM, and IgA by ELISA

IgG, IgM, and IgA were quantified from eluted DBS samples by standard sandwich ELISA methodology (Human IgG, IgM, and IgA ELISA Ready-SET-Go!® Kits, eBioscience, San Diego, CA, USA; Tecan GENios Pro Reader platform, Tecan, San Jose, CA, USA). Analyte-specific capture antibody (anti-human IgG, IgM, or IgA) was bound to a 96-well microtiter plate to create the solid phase matrix. Unbound antibody was removed by washing twice with 400 μL/well of wash buffer (PBS, 0.05 % Tween-20). Plates were blocked with 250 μL of blocking buffer (PBS, 0.10 % Tween-20, 1 % bovine serum albumin) at room temperature for 2 h and then washed twice with 400 μL/well of wash buffer. Following the wash, 100 μL of all calibrators/standards and quality control (QC) samples, and patient samples were added to wells in replicates of two. Plates were then sealed and incubated at room temperature for 2 h with gentle shaking (400 to 450 RPM). Plates were washed 4 times with 400 μL/well wash buffer, and 100 μL/well of horseradish peroxidase conjugated secondary detection antibody was added. Plates were then sealed and incubated at room temperature for 1 h with gentle shaking (400 to 450 RPM). Plates were washed 4 times with 400 μL/well wash buffer, and 100 μL of tetramethylbenzidine substrate solution was added. After the plates were incubated at room temperature for 25 min and the reaction was stopped with 100 μL 2 N H_2_SO_4_, the optical density (OD) of each well was read at 450 nm, and adjusted by subtracting the OD of the same well at 550 nm. The immunoglobulin concentrations were quantified using an external standard curve (see below).

### Validation for Ig-DBS Assay

Validation experiments were conducted in accordance with College of American Pathologists (CAP) and Clinical Laboratory Improvement Amendments (CLIA) (1988) standards and regulations as well as good clinical laboratory practices.

#### Standard Curve Parameters

An external standard curve for each Ig isotype was constructed using optical densities to quantify the Ig concentration in the DBS eluate in relation to the standard curve. The external standard curves for IgG, IgM, and IgA were developed using 7 standards provided by the manufacturer (eBioscience human IgG, IgM, and IgA ELISA Standards). The standard concentrations specified in the product information ranged from 1.6 to 100 ng/mL (IgG and IgA) and 15.6 to 1000 ng/mL (IgM).

#### Quality Control Samples and Limits of Quantitation

QC samples were prepared by adding IgG, IgM, and IgA (eBioscience human IgG, IgM, and IgA ELISA Standards) to assay diluent at 3 concentrations spanning the range of the standard curve: low-QC, mid-QC, and high-QC (LQC, MQC, and HQC, respectively). Quantitation limits were assessed by analysis of freshly prepared standards in duplicate over 10 independent analytical batches to assess reproducibility and establish limits of quantification. The lower limit of quantitation (LLOQ) and upper limit of quantitation (ULOQ) were defined as the lowest and highest standard concentrations that could be measured within the same limits of precision and accuracy as the standard nephelometry assay (≤25 % coefficient of variation [CV] and ±25 % bias, from the expected value for IgG, and ≤15 % CV and ±15 % bias from the expected value for IgM and IgA). These limits are the Total Allowable Error (TeA) specifications for each of these respective serum analytes on the Siemens Dade Behring BNII instrumentation and nephelometry methodology that was used for correlation during the validation [31–33].

#### Precision and Accuracy

Intra-assay precision, the reproducibility of a measurement of an analyte within a single analytical batch, was determined based on the mean of 20 replicates contained in that particular batch. Inter-assay precision, among multiple analytical batches, was calculated at each level based on the mean of 5 replicates of 5 control concentrations of QC generated over 6 batches. Precision limits of ≤25 % for IgG and ≤15 % for IgM and IgA were used to determine assay acceptability. These limits are the Total Allowable Error (TeA) specifications for each of these respective serum analytes on the Siemens Dade Behring BNII instrumentation and nephelometry methodology that was used for correlation during the validation.

Accuracy, the closeness of a measurement to a known value, was assessed based on comparison of the mean value from the intra-assay batch to the achieved mean of the inter-assay experiment described above for each concentration of QC. Accuracy limits of ±25 % bias for IgG and ±15 % bias for IgM and IgA were used to determine assay acceptability.

#### Specificity and Selectivity of the Assay

Specificity is defined as the assay’s ability to differentiate and quantify the specific immunoglobulin in the presence of other components of the elution buffer. The effect of elution buffer alone and samples with IgG, IgM, or IgA concentrations below LLOQ on the assay method were evaluated by analyzing 6 different preparations of the elution buffer. Any measurable presence of interfering elution buffer components was acceptable if the measurement was ≤20 % of the LLOQ for the analyte and if 80 % of the 6 different preparations met this criterion.

Selectivity of an assay is defined as the ability of the method to differentially measure a particular immunoglobulin isotype in a complex medium without interference by other components of the mixture. To test the selective detection of IgG, IgM, and IgA, the cross-reactivity of the conjugated secondary detection antibody in matrix was analyzed in replicates of 10 for 3 different concentrations of each immunoglobulin sample). The cross-reactivity response was measured and calculated using a standard curve. A signal below LLOQ was considered acceptable for selectivity.

#### Linearity of Dilution

The analytical measurement range (AMR) for the assay was established by dilutional linearity. Linearity was defined as the ability of the assay, within a specified range, to produce results that are directly proportional to the concentration of immunoglobulin in the elution buffer. Dilutional linearity was expressed in terms of % bias, a measure of the agreement between the achieved mean and the expected value. Achieved mean values were compared with the expected values by linear regression and considered acceptable if they were within the criteria of ±2 times the maximum allowable % CV of 25 % for IgG and 15 % for IgA and IgM, following established CAP/CLIA guidelines for these immunoglobulin isotypes.

Two DBS samples for each immunoglobulin isotype, one representing a high serum Ig level (1350 mg/dL for IgG, 275 mg/dL for IgM, and 272 mg/dL for IgA) and the other representing a low serum Ig level (870 mg/dL for IgG, 80.5 mg/dL for IgM, and 164.5 mg/dL for IgA), as determined by the US Food and Drug Administration-approved automated nephelometry assay (Siemens Dade Behring BN II Nephelometer assay), were evaluated for precision and accuracy from 4 replicates of each dilution. The maximum and minimum dilutions for each isotype were established defining the AMR.

### Correlational Studies

The correlational studies compared the immunoglobulin (IgG, IgM, IgA) concentrations obtained using the Ig-DBS assay on DBS eluates with those obtained using the standard nephelometry assay on serum samples from the same patients. A key objective of these studies was to determine whether or not the reference ranges for IgG, IgM, and IgA established with the standard nephelometry assay could also be employed for the Ig-DBS assay.

#### Data Analysis

Results from the two assays were compared and a Pearson correlation equation was developed to convert or normalize the Ig-DBS assay value (which is non-normalized, raw data) to a nephelometry serum-equivalent value. The correlation equation obtained from the measured correlation data for IgG, IgM, and IgA was used to generate the normalized dataset. Results obtained by normalizing the Ig-DBS data were compared with the serum non-normalized data to evaluate for any potential bias (% bias = [Ig-DBS value minus nephelometry value] divided by nephelometry value] multiplied by 100).

Reference ranges for IgG, IgM, and IgA were verified using samples from 40 normal adults who had Ig-DBS values and standard nephelometry serum assay values. The Ig-DBS values were normalized using the Pearson correlation equation, and the normalized values were assessed for whether they were within the reference range for adults.

### Sample Stability

The stability of IgG, IgM, and IgA in the DBS was tested at room temperature, 36° to 38 °C, 2° to 8 °C, and −25 °C to −40 °C, from 4 to 14 days. The DBS samples were enclosed in a polyethylene bag placed in a sealed envelope. Stored samples were compared with those assayed without storage and were considered stable if % bias was ≤25 % for IgG and ≤15 % for IgM and IgA, following established CAP/CLIA guidelines for these immunoglobulin isotypes.

## Results

### Subjects

DBS and serum samples were simultaneously obtained from 107 healthy individuals (50 males and 57 females).

### Validation for Ig-DBS Assay

#### Standard Curve Performance and Limits of Quantitation

The calibration curve for IgG revealed that precision and accuracy remained within ± 24 % for concentrations ranging from 1.6 to 100.0 ng/mL, defining the dynamic range of the assay (i.e., range of antigen concentrations that can be measured accurately), since both concentrations were within the acceptable precision and accuracy parameters (≤25 % CV and ±25 % bias from the expected value for IgG). The IgG LLOQ was set at 3.9 ng/mL. For IgM, the precision and accuracy remained within ±6 % for concentrations ranging from 15.6 to 1000 ng/mL. The IgM LLOQ was set at 49.3 ng/mL. For IgA, the precision and accuracy remained within ±15 % for concentrations ranging from 1.6 to 100.0 ng/mL. This defined the dynamic range of the assay, and the IgA LLOQ was set at 4.5 ng/mL.

#### Precision and Accuracy

Five concentrations of QC controls were analyzed 20 times each in one analysis (for each, *n* = 20) for intra-assay precision. The intra-assay precision for the IgG QC samples ranged from 6.7 to 17.1 %, all values passed the criterion of ≤25 % CV. The respective values for IgM and IgA QC samples were 3.6 to 7.1 % and 2.6 to 3.7 %, all values for precision passed the criterion of ≤15 % CV (Table 1).

**Table 1:** Intra- and inter-assay precision and accuracy for IgG, IgM, and IgA

QC samples	Intra-assay precision (CV%)	Intra-assay accuracy (% bias)	QC samples	Inter-assay precision (CV%)	Inter-assay accuracy (% bias)
IgG			IgG		
LLOQ (3.2 ng/mL)	12.3	−17.1	LLOQ (3.9 ng/mL)	19.1	−16.5
Low QC (7.2 ng/mL)	10.7	−16.7	LQC (8.6 ng/mL)	18.6	−7.7
Middle QC (14.2 ng/mL)	9.5	−8.6	MQC (15.6 ng/mL)	19.9	−16.9
High QC (33.5 ng/mL)	6.7	−2.3	HQC (34.3 ng/mL)	15.2	−8.7
ULOQ (57.7 ng/mL)	17.1	−9.2	ULOQ (63.5 ng/mL)	16.5	−15.3
IgM			IgM		
LLOQ (43.4 ng/mL)	7.1	11.8	LLOQ (49.3 ng/mL)	14.0	5.1
Low QC (91.8 ng/mL)	4.4	−8.7	LQC (100.5 ng/mL)	7.5	7.2
Middle QC (190.8 ng/mL)	4.2	−5.3	MQC (201.6 ng/mL)	5.9	7.5
High QC (371.2 ng/mL)	3.6	−5.4	HQC (392.3 ng/mL)	6.6	4.6
ULOQ (791.8 ng/mL)	4.3	−1.3	ULOQ (802.5 ng/mL)	5.9	7.0
IgA			IgA		
LLOQ (4.0 ng/mL)	3.7	−9.3	LLOQ (4.5 ng/mL)	11.1	−4.9
Low QC (8.5 ng/mL)	2.7	−5.9	Low QC (9.0 ng/mL)	7.0	−3.8
Middle QC (17.0 ng/mL)	2.6	−3.9	Middle QC (17.7 ng/mL)	5.8	−5.5
High QC (35.3 ng/mL)	2.8	−2.7	High QC (36.3 ng/mL)	4.9	−3.3
ULOQ (71.9 ng/mL)	3.5	−1.5	ULOQ (73.0 ng/mL)	4.4	−2.6

*CV* coefficient of variation, *LLOQ* lower limit of quantitation, *QC* quality control, *LQC* low range of the dynamic range QC, *MQC* middle of the dynamic range QC, *HQC* high range of the dynamic range QC, *QC* quality control, *ULOQ* upper limit of quantitation

The intra-assay accuracy for the IgG QC samples ranged from −2.3 to −17.1 %, and all values passed the criterion of ±25 % bias. The respective values for IgM and IgA QC samples were −1.3 to −11.8 % and −1.5 to −9.3 %, and all values passed the criterion of ±15 % bias (Table 1).

Five levels of QC controls were analyzed 6 times (for each, *n* = 5) for inter-assay precision. The inter-assay precision for the IgG QC samples ranged from 15.2 to 19.1 %. All values passed the criterion of ≤25 % CV. The respective values for IgM and IgA QC samples were 5.9 to 14.0 % and 4.4 to 11.1 %, and all values passed the criterion of ≤15 % CV (Table 1).

The inter-assay accuracy for the IgG QC samples ranged from −7.7 to −16.9 %, and all values passed the criterion of ±25 % bias. The respective values for IgM and IgA QC samples were 4.6 to 7.5 % and −2.6 to −5.5 %, and all values passed the criterion of ±15 % bias (Table 1).

#### Analytical Specificity and Selectivity

The specificity and selectivity for IgG, IgM, and IgA are shown in Table 2. IgG, IgM, and IgA levels were all below the LLOQ in empty microtiter plates. Capture of IgG by the anti-IgA and anti-IgM antibodies was below the LLOQ. This was also the case for capture of IgM by anti-IgG and anti-IgA antibodies and capture of IgA by anti-IgG and anti-IgM antibodies.

**Table 2:** Specificity and selectivity of the Ig-DBS assay

IgG								
	Specificity		Selectivity					
			IgG calibrator captured by anti-IgA			IgG calibrator captured by anti-IgM		
	Matrix	IgG added at 3.1 ng/mL	37.5 ng/mL	18.75 ng/mL	9.38 ng/mL	37.5 ng/mL	18.75 ng/mL	9.38 ng/mL
Mean	<LLOQ	1.9	2.6^a^	2.8^a^	2.4^a^	3.2^a^	2.5^a^	2.2^a^
SD	–	0.2	–	–	–	–	–	–
% CV	–	8.9	–	–	–	–	–	–
% accuracy	–	62.3	–	–	–	–	–	–
^a^<LLOQ of 4.7 ng/mL								
IgM								
	Specificity^a^		Selectivity					
			IgM calibrator captured by anti-IgA			IgM calibrator captured by anti-IgG		
	Matrix	IgM added at 15.6 ng/mL	375.0 ng/mL	187.5 ng/mL	93.8 ng/mL	375.0 ng/mL	187.5 ng/mL	93.8 ng/mL
Mean	<LLOQ	<LLOQ	<LLOQ	<LLOQ	<LLOQ	<LLOQ	<LLOQ	<LLOQ
^b^LLOQ = 46.9 ng/mL								
IgA								
	Specificity		Selectivity					
			IgA calibrator captured by anti-IgG			IgA calibrator captured by anti-IgM		
	Matrix	IgA added at 3.1 ng/mL	37.5 ng/mL	18.75 ng/mL	9.38 ng/mL	37.5 ng/mL	18.75 ng/mL	9.38 ng/mL
Mean	<LLOQ	2.8	1.1^a^	1.1^a^	1.1^a^	4.2^a^	2.5^a^	1.8^a^
SD	–	0.1	–	–	–	–	–	–
% CV	–	3.7	–	–	–	–	–	–
% accuracy	–	88.6	–	–	–	–	–	–
^a^<LLOQ of 4.7 ng/mL								

*CV* coefficient of variation, *LLOQ* lower limit of quantitation, *SD* standard deviation

^a^The assay was unable to quantify analyte added to matrix at 15.6 ng/mL as well as in matrix without added analyte, thereby demonstrating specificity

^b^Results in the assay for all concentrations that are below the LLOQ of 46.9 ng/mL for the assay indicate no selectivity issue

#### Linearity of Dilution

A Pearson coefficient of determination (R^2^) of 1.00 between the target dilution concentration and the achieved value was demonstrated over the range evaluated for IgG. Similar results were obtained for IgM and IgA. Percent bias values for IgG ranged from −22 to 12.1 %; those for IgM ranged from −1.7 to 10.2 %; and those for IgA ranged from −23.5 to 22.1 %. All were within the acceptable range of ± 2 times the % CV established for each isotype.

### Correlational Studies

#### Correlations with Non-normalized Data

In this analysis, where *x* is DBS value in ng/mL and *y* is a normalized value in mg/dL, the regression analysis formula for IgG was *y* = 48.962*x* − 17358 (*n* = 101) and *r* = 0.834. For IgM, the regression analysis formula was y = 121.6x + 115.13 (*n* = 107) and *r* = 0.789. The regression analysis formula for IgA was *y* = 38.942x − 2000.8 (*n* = 107) and *r* = 0.918.

#### Correlations with Normalized Data

**Fig. 2:**
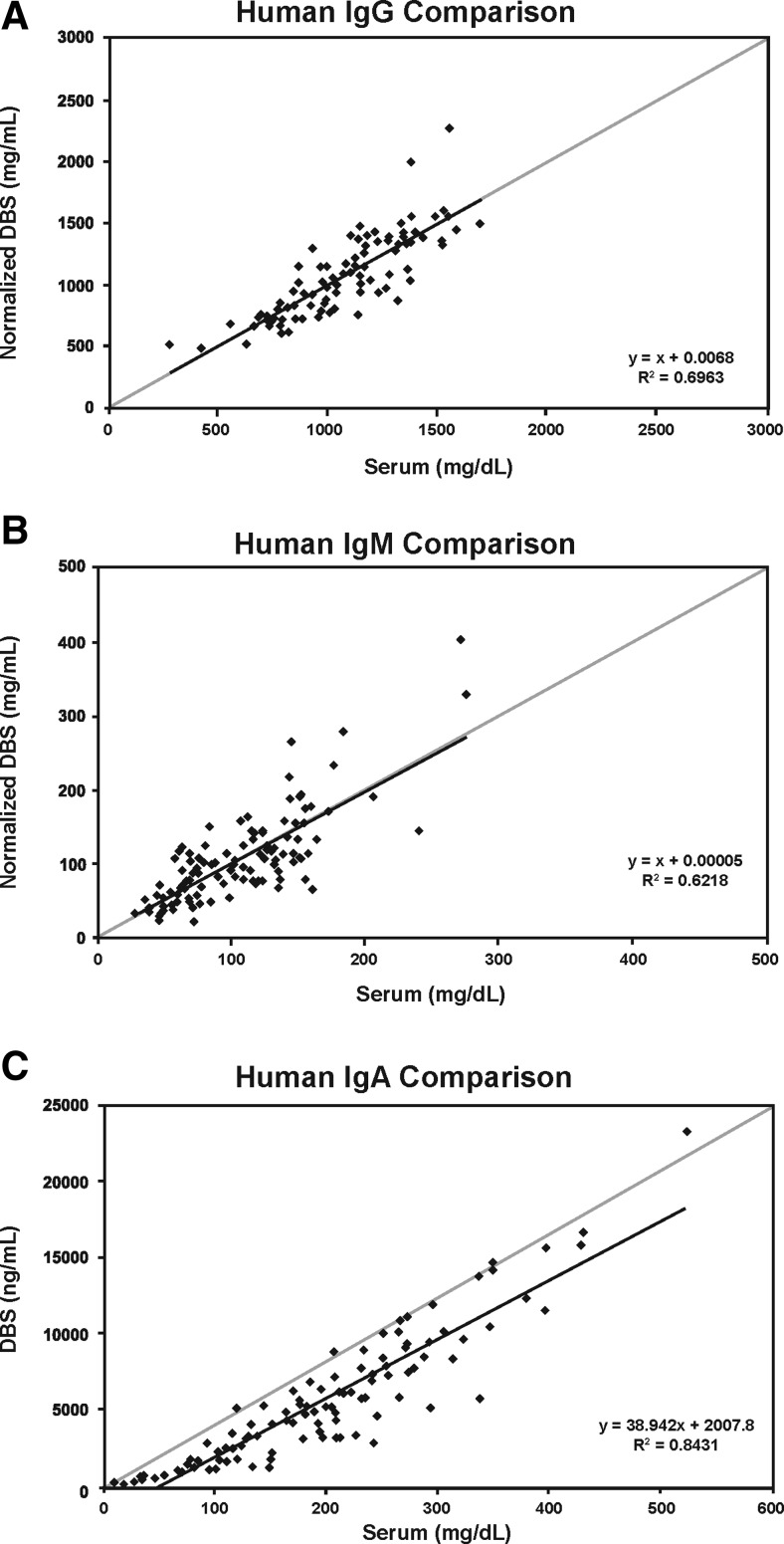
Correlations between standard nephelometry assay and Ig-DBS for IgG (**a**), IgM (**b**), and IgA (**c**) (*scatterplot* and *regression line*)

The normalized values for IgG, IgM, and IgA in mg/dL were calculated using the above regression analysis formulas. Then, regression formulas using the normalized values were established. The regression analysis formula for IgG was *y* = 1.000*x* + 0.0068 (*n* = 101) and *r* = 0.834 (Fig. 2a). The average bias and those at the low and high medical decision level ranges (694 and 1618 mg/dL, respectively) were all 0.0 %. The maximum allowable bias was ±25 %. For IgM, the regression analysis formula was *y* = 1.000*x* − 0.00005 (*n* = 107) and *r* = 0.789 (Fig. 2b). The average bias and those at the low and high medical decision level ranges (48 and 271 mg/dL, respectively) were all 0.0 %. The maximum allowable bias was ±15 %. For IgA, the regression analysis formula was *y* = 1.0001*x* − 0.001 (*n* = 107), and *r* = 0.918 (Fig. 2c). The average bias and those at the low and high medical decision level ranges (81 and 463 mg/dL, respectively) were all 0.0 %. The maximum allowable bias was ±15 %.

#### Reference Ranges

Forty subjects had simultaneous blood draws for serum samples and also provided samples for DBS. The IgG, IgM, IgA tests were run on all 40 samples in order to verify that the reference ranges used by Quest Diagnostics and other laboratories for the nephelometry assay are applicable to this normal population. Determination of whether the reference ranges for the standard nephelometry serum assay could also be applied to the Ig-DBS was accomplished by comparing numbers of results outside the reference ranges for IgG, IgM, and IgA for each of the assays. The reference range for IgG in male and female individuals ≥16 years of age was 694 to 1618 mg/dL. Results for 39 of the 40 subjects were within this range for both the standard nephelometry and Ig-DBS assays. The reference range for IgM in male and female individuals ≥16 years of age was 48–271 mg/dL. Results for 35 of 40 subjects were within this range for both the standard nephelometry and Ig-DBS assays. When the DBS values were converted or normalized to serum mg/dL values, 4 serum and 4 DBS normalized results independent of the source individual were outside the reference range (1 above and 3 below). The reference range for IgA in male and female individuals ≥16 years of age is 81 to 463 mg/dL.

For IgG, the LLOQ and ULOQ of the Ig-DBS assay were established at 3.9 ng/mL and 63.5 ng/mL, respectively. This result, combined with the established linearity of dilution of DBS samples and the correlation equations derived from the DBS and serum assays, generated a normalized low of 370 mg/dL and a high of 6832 mg/dL for the reportable values range, respectively. The reference range for IgG in the standard nephelometry assay for males and females in the age range ≥16 years was 694 to 1618 mg/dL and fell within these limits. For IgM, the LLOQ and ULOQ of the Ig-DBS assay were established at 49.3 ng/mL and 802.5 ng/mL, respectively. Combined with the established linearity of dilution for DBS samples, this generated a normalized low and high of 9 and 6581 mg/dL for the reportable values range, respectively. The reference range for IgM in the standard nephelometry assay for males and females in the age range ≥16 years was 48 to 271 mg/dL and was within these limits. For IgA, the LLOQ and ULOQ of the Ig-DBS assay were established at 4.5 ng/mL and 73.0 ng/mL, respectively. Combined with the established linearity of dilution of DBS samples, this resulted in a normalized low of 63 mg/dL and a high of 3804 mg/dL for the reportable values range, respectively. The reference range for IgA in the standard nephelometry assay for males and females ≥16 years of age was 81 to 463 mg/dL, and it also fell within the limits for the Ig-DBS assay.

All these results indicate that the reference ranges for IgG, IgM, and IgA established for individuals ≥16 years of age with the standard nephelometry assay can also be used for the normalized values derived from the Ig-DBS assay. The current study focused on establishing the IgDBS assay for individuals ≥ 16 years of age since the normal *adult* reference ranges apply to this group; which made it possible to evaluate the assay without the added complexity of age-adjusted normal values that are typically lower in younger children. Sample results from younger children were used only as anchor points for establishment of the regression equations at the lower end of the range.

### Stability of DBS

When stored in a polyethylene bag, IgG, IgM, and IgA were stable (≤25 % bias for IgG and ≤15 % bias for IgM and IgG) at room temperature and at 2° to 8 °C for 14 days. At 36° to 38 °C, IgG and IgM were stable for 4 days and IgA was stable for 3 days. At −25 °C to −40 °C, IgG and IgM were stable for 14 days and IgA was stable for 10 days (Table 3).

**Table 3:** Days of stability of IgG, IgM, and IgA in DBS^a^

Analyte	Room temperature	2 to 8 °C	36 to 38 °C	−20 to −40 °C
IgG	14	14	4	14
IgM	14	14	4	14
IgA	14	14	3	10

^a^Storage in polyethylene bag

Maximum number of days evaluated at room temperature = 14; at 2°–8 °C = 14; at 36°–38 °C = 4; at −20° to −40 °C = 14

## Discussion

Based on the fact that there remains significant diagnostic delay in identifying patients with hypogammaglobulinemia [6, 11] there is a need for a convenient, reliable and targeted method to evaluate circulating immunoglobulin levels in order to decrease the threshold to screen for antibody deficiency. Ideally, the screening tool needs to be convenient, simple to use, and able to capture the patient immediately at the site of care. It should also be usable in a variety of clinical settings to maximize utility as a potential screening tool for hypogammaglobulinemia. We believe that a simple method that allows sample collection when patients are physically in the clinic will decrease the threshold to screen patients for antibody deficiencies, thereby potentially shortening the delay to diagnosis that is common for these disorders.

Patients with PID sometimes are not diagnosed until they are hospitalized for a life-threatening infection [34]. This was highlighted in a survey of nearly 1300 patients with PID carried out by the Immune Deficiency Foundation. The survey showed that the average duration from symptom onset to diagnosis was 12.4 years for all types of PID, 4.4 years for agammaglobulinemia, and 14 years for common variable immunodeficiency (CVID) [35]. A survey by the Primary Immunodeficiency Association indicated that 57 % of patients in the United Kingdom had to see their primary care physician more than 10 times before being referred to a specialist; for 25 % of adult patients, the time from initial presentation to diagnosis of PID was greater than 7 years [36]. A study of patients with CVID also indicated a mean diagnostic delay of 7 years [37].

DBS samples have been successfully used in various newborn screening tests of genetic diseases for many years. More recently, DBS have been demonstrated to be a convenient blood sample collection device for T cell receptor excision circle (TREC) analysis in SCID screening [20, 38, 39] and for determination of C3 deficiency [40]. Analysis of kappa-deleting recombination excision circles (KREC) using DBS samples has also been developed [25]. The applicability of these assays is however limited to patients with severe T or B cell lymphopenia due to defective recombination of the T or B cell receptor gene locus.

This approach is designed to detect severe immunodeficiencies, many of which are attributable to distinct immunologic and/or genetic mechanisms but it fails to detect the majority of patients who ultimately develop antibody deficiency. The testing platform presented here extends the utility of DBS to quantification of IgG, IgM, and IgA as a screening tool for hypogammaglobulinemia in patients 16 years of age and older. We predict that this method will also serve as a screening tool for younger children as well but we have not yet evaluated enough samples from pediatric patients of various age groups to perform adequate validation testing.

There are several advantages of DBS that make this a valuable testing platform for hypogammaglobulinemia screening. First, it allows collection at the point of care or even in a patient’s home, which should decrease barriers to having testing performed. Second, it could facilitate rapid, efficient screening of large groups of high-risk individuals at patient or family gatherings. Third, shipping of the card via standard mail, which is much cheaper than sending a liquid blood sample, would facilitate use of this screening test in rural areas or in developing countries where timely shipment of liquid blood samples can be a logistical challenge. Although the DBS described has substantial utility, it should be kept in mind that it is not designed to detect forms of PID that are not associated with decreased Ig levels (e.g., complement deficiency and phagocyte defects) [30]. Thus, normal DBS results should not eliminate the possibility of a PID in a patient with clinical symptoms that would prompt suspicion of this diagnosis.

The present study was carried out to validate the collection of DBS with analysis by ELISA for quantification of serum IgG, IgM, and IgA using standardized experimental samples and serum from individuals expected to have normal immunoglobulin levels. ELISA for measurement of immunoglobulins is much more sensitive than the conventional nephelometry assay, thereby allowing the use of very small quantities of specimen [41–43]. In addition, good correlations between protein concentrations measured with ELISA and nephelometry have been shown in previous studies comparing the two methods [44, 45]. The Ig-DBS assay we developed demonstrated precision, accuracy, specificity, selectivity, and linearity. The correlation between the Ig-DBS assay and standard nephelometry assay was verified with high correlation coefficients of 0.834 for IgG, 0.789 for IgM, and 0.918 for IgA. It was also determined that normal reference ranges for all 3 serum Ig isotypes from the standard nephelometry assay could be used with the Ig-DBS assay normalized values in individuals ≥16 years of age. Accordingly, the Ig-DBS assay had sufficient clinically reportable ranges (CRR), particularly at the low end, i.e. 370 mg/dL for IgG; 9 mg/dL for IgM; and 63 mg/dL for IgA, to provide a suggestion for comprehensive immunological evaluation. The Ig-DBS results for all 3 serum Ig isotypes are reported as concentrations in mg/dL with normal reference ranges. Further costs associated with phlebotomy and shipping of blood tubes are not required for blood sampling by DBS and thus should be considered as reducing the overall cost of a DBS-based approach.

Lastly, we assessed the stability of IgG, IgM, and IgA in DBS samples stored in various conditions, information critical to understanding whether this approach would be stable enough to allow for shipping of samples at ambient temperature to a centralized laboratory for processing and measurement. Previous studies of the stability of large biologic molecules in DBS have demonstrated stability for more than 1 week across a wide range of temperatures [46–48]. However, it has also been shown that extreme storage conditions, including high humidity, very high temperature (55 °C), and exposure to daylight for long periods can decrease recovery of large molecules from DBS [49]. Results of this study showed that DBS samples were stable over 14 days at room temperature, 3 days at 36°–38 °C, 14 days at 2°–8 °C, and ≥10 days at −25° to −40 °C in a sealed polyethylene bag. Therefore, the Ig-DBS assay should enable transportation of the samples at room temperature via regular mail.

In conclusion, the Ig-DBS assay is both sensitive and accurate for quantification of serum immunoglobulins with convenient sample collection and shipment. This assay thus presents a potential new alternative for screening patients with suspected hypogammaglobulinemia. As with any screening test, further laboratory evaluation, including repeated measurement of serum Ig levels using standard nephelometry assay in patients with Ig levels at the lower end of the normal range, and referral to a specialist would be recommended for definitive diagnosis.
